# Data supporting the effects of lysozyme on mRNA and protein expression in a colonic epithelial scratch wound model

**DOI:** 10.1016/j.dib.2016.12.043

**Published:** 2016-12-29

**Authors:** Sarah K. Abey, Yuana Yuana, Paule V. Joseph, Natnael D. Kenea, Nicolaas H. Fourie, LeeAnne B. Sherwin, Gregory E. Gonye, Paul A. Smyser, Erin S. Stempinski, Christina M. Boulineaux, Kristen R. Weaver, Christopher K.E. Bleck, Wendy A. Henderson

**Affiliations:** a Digestive Disorders Unit, Division of Intramural Research, National Institutes of Health, Department of Health and Human Services, Bethesda, MD, USA; b Image Sciences Institute, Division of Imaging, University Medical Centre Utrecht, Netherlands; c NanoString Technologies, Seattle, WA, USA; d The Pennsylvania State University, College of Medicine, Hershey, PA, USA; e Electron Microscopy Core Facility, National Heart, Lung, and Blood Institute, National Institutes of Health, Department of Health and Human Services, Bethesda, MD, USA

## Abstract

Colonic epithelial health is implicated in a host of gastrointestinal (GI) diseases and disorders. Lysozyme is suspected to play a role in the ability of the epithelium to recover from injury (Abey et al., in press; Gallo, 2012; Rubio, 2014) [1], [2], [3]. Disrupted repair mechanisms may lead to delayed or ineffective recovery and disruptions to epithelial biology resulting in GI symptoms and altered barrier function (Peterson and Artis, 2014) [4]. The effect of lysozyme on the transcriptomic and proteomic profile of healthy colonic epithelial cells was investigated. Epithelial cells in culture were scratch wounded and treated with lysozyme. mRNA and protein profiles were simultaneously quantified in the same sample using a digital counting technology. Gene and protein expressions altered by the presence or absence of lysozyme are described in this article. Extensive statistical and bioinformatic analysis, and interpretation of the results can be found in “Lysozyme association with circulating RNA, extracellular vesicles, and chronic stress” (Abey et al., in press) [1].

**Specifications Table**

**Table:** 

Subject area	*Health Sciences*
More specific subject area	*Cellular wound healing*
Type of data	*Tables and figure*
How data was acquired	*Human fetal colon epithelial cell line CRL-1790 (ATCC, Manassas, VA)*
	*nCounter PanCancer Immune Panel (NanoString)*
Data format	*Analyzed*
Experimental factors	*Lysozyme*
Experimental features	*Selected mRNA and proteins were barcode labeled directly in the cell lysate and simultaneously digitally counted*
Data source location	*National Institutes of Health, Bethesda, MD, USA*
Data accessibility	*Within this article*
Related research article	*Abey et al., (2016). “Lysozyme association with circulating RNA, extracellular vesicles, and chronic stress”* BBA-Clinical

**Value of the data**

• These data describe changes in immune and inflammatory-relevant mRNAs and proteins during cellular wound healing in the presence of lysozyme.
• Data show cellular reprograming in epithelial cells that survive scratch wounding.
• Data show the effect of lysozyme on wound healing in cultured cells.

## 1 Data

**Fig. 1:**
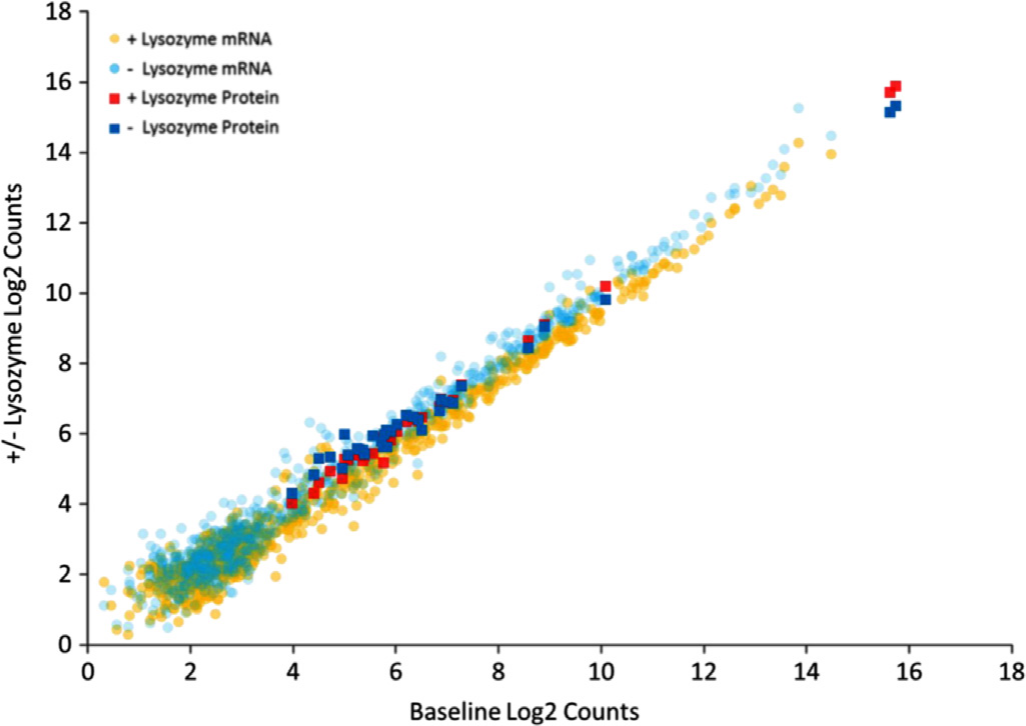
mRNA and protein counts of scratch wounded healthy colonic epithelial cultures with and without lysozyme (y-axis, log2 scale) plotted against baseline (x-axis, log2 scale).

The fold changes in gene and protein expression as a result of the scratch wound and/or the addition of lysozyme is presented in Table 1. Fig. 1 shows simultaneous changes in gene and protein expression in cells that survive wounding in the presence or absence of lysozyme.

## 2 Experimental design, materials and methods

Lysozyme, found in circulating plasma EVs [1], is an antimicrobial agent [2] thought to contribute to the biological responses to injury within the intestinal epithelium [3-4]. Healthy colonic epithelial cells were cultured in the lab and subjected to a scratch assay to examine the effects of lysozyme on wound healing [1] . Resulting transcriptomic (770 genes) and proteomic (30 proteins) changes in the cultures were simultaneously measured using a digital counting assay.

### 2.1 Cell culture

Human fetal colon epithelial cells (CRL-1790, ATCC, Manassas, VA) were maintained in Eagle׳s Essential Minimal Media (EMEM) supplemented with 10% fetal bovine serum (ATCC) in a humidified 5% CO_2_ atmosphere at 37 °C in an incubator.

### 2.2 Wound healing assay

Cells were grown in duplicates in 6-well plates (7×10^5^/well). Once confluent, the cultures were pre-treated with serum-free media containing 200 µg/mL Bovine Serum Albumin (BSA; New England Biolabs, Ipswich, MA) for 12 h. The following day, the monolayers were scratched using a sterile micropipette tip. The scratched monolayers were then washed with serum-free media, fresh serum-free media containing only BSA (“serum-free”), or BSA with purified chicken white egg lysozyme (10 µg/mL; Sigma-Aldrich, St. Louis, MO). Baseline measurements were taken at the 0 h time point. At 8 h, post-treatment migration into scratch wound gaps was observed in lysozyme-treated cultures. Three biological replicates were collected from three separate days of experiments.

### 2.3 Simultaneous protein and mRNA quantification

Cells were collected and stored in cryoprotected media at −80 °C. The simultaneous measurement of immune relevant mRNA and protein profiles were done using the nCounter RNA: Protein PanCancer Immune Panel (NanoString, Seattle, WA). Cells were thawed and prepared as per the specifications of the nCounter© assay. The prepared lysates were assayed as per the assay protocol without modification. Data processing, QC, and normalization were done as per the manufacturer׳s recommendations.
